# Systemic juvenile idiopathic arthritis in the republic of Bashkortostan

**DOI:** 10.1186/1546-0096-9-S1-P146

**Published:** 2011-09-14

**Authors:** VA Malievskiy

**Affiliations:** 1 Bashkir State Medical University, Ufa, Russia

## Background

Juvenile idiopathic arthritis (JIA) is the most common rheumatic disease in children. In the structure of JIA, systemic arthritis makes 10 – 15 %. Being one of the variants of JIA it is defined as a heterogeneous disease.

## Aim

To analyze the course, treatment and outcome of systemic JIA in children of the Republic of Bashkortostan (Russia).

## Methods

We observed 56 patients, who fell ill in the age of 16, and were diagnosed systemic JIA. The diagnosis was defined by the ILAR criteria (Edmonton, 2001) as arthritis and a documented quotidian fever of at least two week duration, plus one or more of the following: typical rash, generalized lymphadenopathy, enlargement of liver or spleen, serositis. According to the course of disease the patients were divided into three subgroups:

1. Monocyclic disease course, patients had only one attack.

2. Polycyclic disease course was characterized by recurrent episodes of remission without medication.

3. Chronic persistent course, patients had extended poly-arthritis with or without systemic features.

## Results

We observed 33 boys and 23 girls. Mean age of the patients was 14.5 years (range 2.1-22.3 years). The mean age at diagnosis was 4.9 years (range 0.9-14.2 years). Mean disease duration was 9.6 years. 12 (21.4%) patients had monocyclic disease course, 15 patients (26.8%) had polycyclic disease course, and 29 patients (51.8%) had chronic persistent course. All the patients took non-steroidal anti-inflammatory drugs and oral prednisolone (0.7 – 1.0 mg/kg/day). 41 patients (73.2%) took intravenous methylprednisolone (20 -30 mg/kg/day) during three consecutive days. All the patients took disease-modifying anti-rheumatic drugs, including methotrexate (100 %) and cyclosporine A (17 patients, 30.3 %). Anti-TNF agents were used in three patients, but without any effect. In four patients some positive effect was reached by taking tocilizumab. Complications: structural injury (30 cases, 53.6%), growth retardation (22 cases, 39.3%), osteoporosis (36 cases, 64.3%), amiloidosis (3 cases, 5.3%).

## Conclusion

Despite of similarity of clinical manifestations in debut of systemic arthritis in most patients, disease course considerably differs on different patients. Prognosis and outcome of disease may vary from “recovery” to “hard life-threatening after-effect”.

